# Restricted expression of classic cadherins in the spinal cord of the chicken embryo

**DOI:** 10.3389/fnana.2014.00018

**Published:** 2014-03-31

**Authors:** Juntang Lin, Congrui Wang, Christoph Redies

**Affiliations:** ^1^Institute of Anatomy I, University of Jena School of Medicine – Jena University HospitalJena, Germany; ^2^Xinxiang Medical UniversityXinxiang, Henan, China

**Keywords:** cadherin, spinal cord, motor neuron, cell adhesion, development, gene expression

## Abstract

Classic cadherins belong to the family of cadherin genes and play important roles in neurogenesis, neuron migration, and axon growth. In the present study, we compared the expression patterns of 10 classic cadherins (Cdh2, Cdh4, Cdh6, Cdh7, Cdh8, Cdh9, Cdh11, Cdh12, Cdh18, and Cdh20) in the developing chicken spinal cord (SP) by *in situ* hybridization. Our results indicate that each of the investigated cadherins exhibits a spatially restricted and temporally regulated pattern of expression. At early developmental stages (E2.5–E3), Cdh2 is expressed throughout the neuroepithelial layer. Cdh6 is strongly positive in the roof plate and later also in the floor plate. Cdh7, Cdh11, Cdh12, and Cdh20 are expressed in restricted regions of the basal plate of the SP. At intermediate stages of development (E4–E10), specific expression profiles are observed for all investigated cadherins in the differentiating mantle layer along the dorsoventral, mediolateral, and rostrocaudal dimensions. Expression profiles are especially diverse for Cdh2, Cdh4, Cdh8, Cdh11, and Cdh20 in the dorsal horn, while different pools of motor neurons exhibit signal for Cdh6, Cdh7, Cdh8, Cdh9, Cdh12, and Cdh20 in the ventral horn. Interestingly, subpopulations of cells in the dorsal root ganglion express combinations of different cadherins. In the surrounding tissues, such as the boundary cap cells and the notochord, the cadherins are also expressed differentially. The highly regulated spatiotemporal expression patterns of the classic cadherins indicate that these genes potentially play multiple and diverse roles during the development of the SP and its surrounding tissues.

## INTRODUCTION

As part of the central nervous system (CNS), the spinal cord (SP) plays a role in the transmission of neural signals between the brain and the body; it also contains neural circuits, which control numerous reflexes (Standring, 2008). The vertebrate SP is generated from the caudal neural tube and differentiates along the dorsoventral, lateromedial, and rostrocaudal axes (Edlund and Jessell, 1999; Bellairs and Osmond, 2005). The chicken embryo, an important model species for evolutionary and developmental biology, is well suited for studying SP development and differentiation. The SP of the chicken embryo consists of two layers at 2.5 days of incubation (E2.5; stage 17): the neuroepithelial layer (NE), which lines the central canal and contains a large number of mitotic cells, and the marginal layer of the neural tube, where post-mitotic neuroblasts differentiate into neurons. The (superficial) mantle layer (ML) becomes morphologically visible at E3 (stage 20) and gray and white matter (WM) can be distinguished at E4 (stage 24). The dorsal (DO) and ventral horns (VHs) can be discerned in the gray matter (GM) from about E6. Also, different motor neuron pools are distinguishable at this stage. At E8, the overall structure of the SP resembles that of late stages (Bellairs and Osmond, 2005). Many genes, such as bone morphogenetic proteins, semaphorins, Eph/ephrins, cadherins, and protocadherins are involved in the development of the SP and its associated ganglia (Vleminckx and Kemler, 1999; Krull, 2001; Graham, 2003; Luo et al., 2009; Lin et al., 2010, 2012).

Our group has recently analyzed the expression of eight delta-protocadherins (delta-Pcdhs) during SP development, and found that each of the investigated genes exhibits a spatially restricted and temporally regulated expression pattern in the chicken embryo. Specifically, delta-Pcdhs are regionally expressed in the VH, where distinct motor neuron pools are positive for different cadherins (Lin et al., 2012). Apart from delta-Pcdhs, classic cadherins are another subgroup of the cadherin family of adhesion molecules. They are typically classified into type I and II classic cadherins. Type I classic cadherins, which include *E*-cadherin (Cdh1), *N*-cadherin (Cdh2), *P*-cadherin (Cdh3), and *R*-cadherin (Cdh4) possess the conserved histidine-alanine-valine (HAV) amino acid sequence in their first extracellular cadherin repeat (Takeichi, 1995). Type II classic cadherins have a structure similar to that of the type I proteins, but they do not contain the HAV motif. Most classic cadherins exhibit temporally and spatially distinct expression patterns during embryonic development. The functions of many classic cadherins have been studied in different species and in various tissues. For example, in the nervous system, Cdh2 and Cdh4 proteins are expressed along the neurites of the developing chicken brain, SP and retina and mediate axon elongation by a homotypic binding mechanism (Matsunaga et al., 1988; Redies et al., 1993; Treubert-Zimmermann et al., 2002). Cdh2 is likely to be the only classic cadherin expressed by astrocytes (Ferguson and Scherer, 2012). Similar to some transcription factors, type II classic cadherins are markers for specific motor neuron pools in the SP (Price et al., 2002). Cadherin-7 (Cdh7) and cadherin-6B (Cdh6) differentially regulate the growth, branching, and guidance of neurites at early and late phases of cranial motor neuron development (Barnes et al., 2010). Cdh19 is expressed not only by Schwann cells and their precursors throughout development but also by oligodendrocytes at the prehatching stage (Lin et al., 2010). There is evidence that classic cadherins are involved in the morphogenesis and functional differentiation of the brain and SP (Redies, 2000; Takeichi, 2007; Hirano and Takeichi, 2012). However, to date, the expression of multiple classic cadherins in the developing SP has not been mapped in detail in a comparative study. Therefore, in the present study, we compared the expression patterns of 10 classic cadherins (Cdh2, Cdh4, Cdh6, Cdh7, Cdh8, Cdh9, Cdh11, Cdh12, Cdh18, and Cdh20) in the developing chicken SP by *in situ* hybridization.

## MATERIALS AND METHODS

### PREPARATION OF EMBRYOS

Fresh fertilized eggs of White Leghorn chicken (*Gallus gallus*) were purchased from a local farm and incubated at 37.5°C in a forced-draft incubator (Ehret, Emmendingen, Germany) with 55–65% humidity. We studied at least three embryos each at the following days of incubation (E): E2.5 [stage 17 after Hamburger and Hamilton (1951)], E3 (stage 20), E4 (stage 24), E6 (stage 28), E8 (stage 34), and E10 (stage 36).

After removal from the eggs, embryos were fixed in ice-cold formaldehyde solution (4% in Hepes-buffered salt solution; HBSS, pH7.4) for 6–24 h, depending on the size of the embryos. Following immersion in a series of graded sucrose solutions (12, 15, and 18% in HBSS), specimens were embedded in Tissue-Tek O.C.T. Compound (Science Services, München, Germany). Specimens were frozen in liquid nitrogen and stored at -80°C, as described previously (Lin et al., 2008). All experiments were carried out in accordance with national and institutional guidelines for the use of animals in research. On a refrigerated microtome (Cryo-Star HM560, Microm International, Walldorf, Germany), we obtained series of 20 μm-thick consecutive sections.

### PROBE SYNTHESIS AND *IN SITU* HYBRIDIZATION

**Table 1** lists the recombinant plasmids that contained the cDNA fragments of the 10 investigated classic cadherins. The digoxigenin-labeled sense and antisense cRNA probes were synthesized *in vitro* by using T3, T7, or Sp6 RNA polymerase, according to the manufacturer’s instructions (Roche, Mannheim, Germany). Sense cRNA probes were used as a negative control. Probes were purified by sodium acetate or LiCl/ethanol precipitation and by using Quick Spin Columns (Roche). We verified the correct size and the incorporation of digoxigenin into the cRNA probes by formaldehyde denaturing gel electrophoresis and blotting; membrane-bound probes were detected with alkaline phosphatase-conjugated anti-digoxigenin Fab fragments (Roche).

**Table 1:** Information on the cRNA probes of the 10 investigated classic cadherins.

Cadherin (Cdh)	Other name	Type	Length of cDNA (insert) (bp)	Accession no.	cRNA probe synthesis
Cdh2	Neural cadherin (Ncdh)	Type I	3203(2739)	NM_001001615.1	*Sal*I/T3(*Xba*I/T7)
Cdh4	Retina cadherin (Rcdh)	Type I	2954(2742)	NM_001004391.1	*Hind*III/T3(*Xba*I/T7)
Cdh6	Fetal kidney-cadherin (Kcdh)	Type II	2684(2373)	NM_001001758.1	*Sal*I/T3(*Xba*I/T7)
Cdh7	–	Type II	2920(2358)	NM_204187.2	*Sal*I/T3(*Xba*I/T7)
Cdh8	–	Type II	3676(2400)	NM_001100289.1	*Xba*I/Sp6(*Kpn*I/T7)
Cdh9	T1-cadherin	Type II	3542(2370)	XM_001231540.3	*Xba*I/Sp6(*Kpn*I/T7)
Cdh11	Osteoblast cadherin (OB-cdh)	Type II	5423(2379)	NM_001004371.1	*Xba*I/Sp6(*Kpn*I/T7)
Cdh12	*N*-cadherin-2; Br-cadherin	Type II	3136(2409)	XM_418999.4	*BamH*I/T7(*Xho*I/Sp6)
Cdh18	Cadherin-14	Type II	5273(2372)	XM_426046.4	*Xba*I/Sp6(*Kpn*I/T7)
Cdh20	MN-cadherin	Type II	3172(2397)	NM_204134.1	*Xba*I/Sp6(*Kpn*I/T7)

*For example, Cdh2 (also called neural cadherin or Ncdh) belongs to the type I classic cadherins. The total cDNA length is 3203 bp and the length of the insert in the recombinant cloning plasmid is 2739 bp. The reference accession number for the NCBI database is NM_001001615.1. The plasmid containing the Cdh2 insert is linearized with the restricted enzyme *Sal*I and transcribed *in vitro* into cRNA with RNA polymerase T3 to obtain antisense probe; the information in parentheses is for synthesis of sense probe.*

For *in situ* hybridization on cryosections, we followed the protocol by Lin et al. (2008). In brief, 20 μm-thick cryostat sections were fixed with 4% formaldehyde in phosphate-buffered saline (PBS, pH 7.4). Following pretreatment with proteinase K and acetic anhydride, sections were hybridized with cRNA probe at 70°C overnight at a concentration of 3–5 ng per microliter of hybridization solution (50% formamide, 1x Denhardt’s solution, 3x SSC, 250 μg/ml salmon sperm DNA and 250 μg/ml yeast transfer RNA). The sections were washed and the unbound cRNA probe was removed by RNAse, followed by incubation with alkaline phosphatase-coupled anti-digoxigenin Fab fragments (Roche) at 4°C overnight. In order to visualize the labeled mRNA, sections were incubated with substrate solution (5-bromo-4-chloro-3-indoyl phosphate and nitroblue tetrazolium salt) until enough color precipitate had formed.**

### DOUBLE STAINING FOR *IN SITU* HYBRIDIZATION AND IMMUNOSTAINING

After *in situ* hybridization, immunostaining was performed on the same sections, following a modified version of the protocol described by Lin et al. (2010). In brief, after *in situ* hybridization, the slides were washed three times in Tris-buffered saline (TBS, pH7.4) and fixed in 4% formaldehyde for 20 min. Sections were then washed again in TBS three times, pretreated with blocking buffer (5% milk and 0.3% Triton X-100 in TBS) and incubated overnight at 4°C with primary antibodies against either Islet1 or MNR2 in blocking buffer at a dilution of 1:20. The secondary antibody (goat anti-mouse immunoglobulin Cy3-labeled IgG; Jackson ImmunoResearch) was applied for 1 h at a dilution of 1:300 in blocking buffer at room temperature. To visualize nuclei, the sections were counterstained with Hoechst 33258 (Molecular Probes, Eugene, OR, USA) for 5 min and then covered with Moviol.

### PRIMARY ANTIBODY CHARACTERIZATION

Antibody against Islet1 (39.4D5, Developmental Studies Hybridoma Bank [DSHB], University of Iowa, USA) recognizes a Lim homeodomain protein that is strongly expressed in the medial part of the lateral motor column (LMC) neuron of the SP but weakly in the medial motor column (MMC) neuron (Pfaff et al., 1996). Antibody against MNR2 (81.5C10, DSHB; Tanabe et al., 1998; William et al., 2003) recognizes a member of an evolutionarily conserved subgroup of Mnx class proteins. MNR2 is strongly expressed in the medial MC neuron but more weakly in the lateral MC neuron of the SP (Ferrier et al., 2001). Lin et al. (2012) discussed the specificity of the antibodies in detail.

### PHOTOGRAPH PRODUCTION

Stained sections were viewed and photographed under a transmission microscope (BX40; Olympus, Hamburg, Germany) equipped with a digital camera (DP70; Olympus). Using the Photoshop image processing software (Adobe, Mountain View, CA, USA), images were adjusted in contrast and brightness. To display results for stains simultaneously (**Figures 9** and**10**), photographic images were enhanced in contrast, color coded and superimposed with the Photoshop program.

For neuroanatomical orientation, SP sections were obtained adjacent to the immunostained sections and stained with hematoxylin and eosin (HE). For terminology and identification of neuroanatomical structures, we consulted the atlas by Bellairs and Osmond (2005) and compared our results to the expression patterns of delta-Pcdhs in the developing SP (Lin et al., 2012).

## RESULTS

**FIGURE 1:**
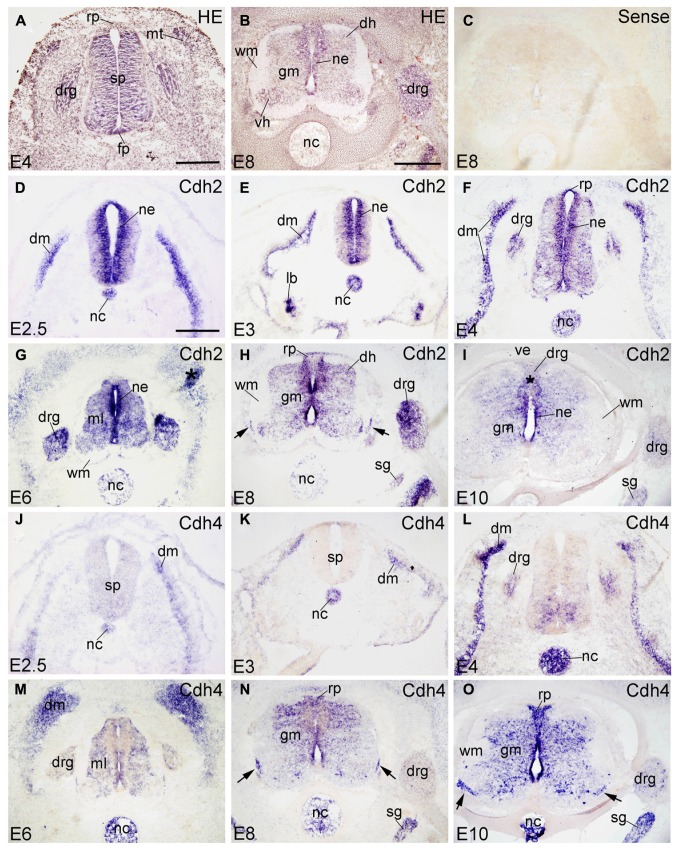
**Expression of Cdh2 and Cdh4 in transverse sections of the developing spinal cord at the lumbar level from 2.5 days’ incubation (E2.5) to E10 (indicated in each panel).** **(A,B)** Hematoxylin and eosin (HE) staining. **(C)** *In situ* hybridization result for sense cRNA-probe used as a negative control. **(D–I)** *In situ* hybridization result for *N*-cadherin (Cdh2) and **(J–O)** for *R*-cadherin (Cdh4). The arrows in **H,N,O** point to the positive boundary cap cells at the position of the ventral root. The asterisks in **G,I **indicate artificial folds in the tissue. Abbreviations: DM, dermomyotome; DH, dorsal horn; DRG, dorsal root ganglion; FP, floor plate; GM, gray matter; ML, mantle layer; MT, myotome; NC, notochord; NE, neuroepithelial layer; RP, roof plate; SG, sympathetic ganglion; SP, spinal cord; VE, vertebral arch; WM, white matter. Scale bars: 100 μm in **D** (for **D,J**); 200 μm in **A** (for **A,E,F,K,L**); 500 μm in **B** (for **B,C,G–I,M–O**).

HE-stained transverse sections revealed the general morphology of the SP and the surrounding tissues; examples are shown for E4 and E8 embryos in **Figures 1A,B**. Sense probes were used as a negative control and did not result in any significant staining of the sections. As a representative example, a section hybridized with sense probe for Cdh2 is displayed in **Figure 1C**.

**FIGURE 2:**
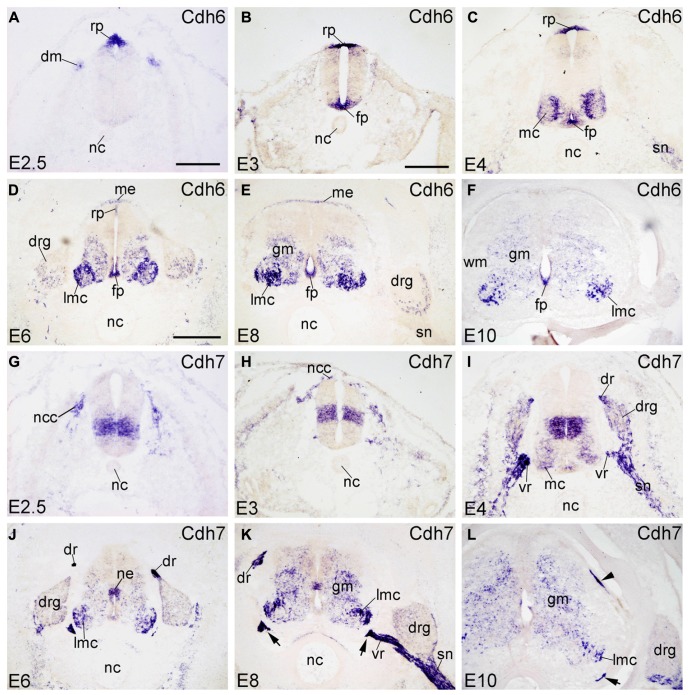
**Expression of Cdh6 and Cdh7 in transverse sections of the developing spinal cord at the lumbar level from 2.5 days’ incubation (E2.5) to E10 (indicated in each panel).** **(A–F)** *In situ* hybridization result for cadherin-6B (Cdh6), and **(G–L) **for cadherin-7 (Cdh7). The arrows in **K,L** point to the positive boundary cap cells at the positions of the ventral root. The arrowhead in **L** points to the positive boundary cap cells at the position of the dorsal root. Abbreviations: DM, dermomyotome; DR, dorsal root; DRG, dorsal root ganglion; FP, floor plate; GM, gray matter; MC, motor column; ME, meninges; ML, mantle layer; NC, notochord; NCC, neural crest cell; NE, neuroepithelial layer; RP, roof plate; SN, spinal nerve; VR, ventral root; WM, white matter. Scale bars: 100 μm in **A** (for **A,G**); 200 μm in **B** (for **B,C,H,I**); 500 μm in **D** (for **D–F,J–L**).

**FIGURE 3:**
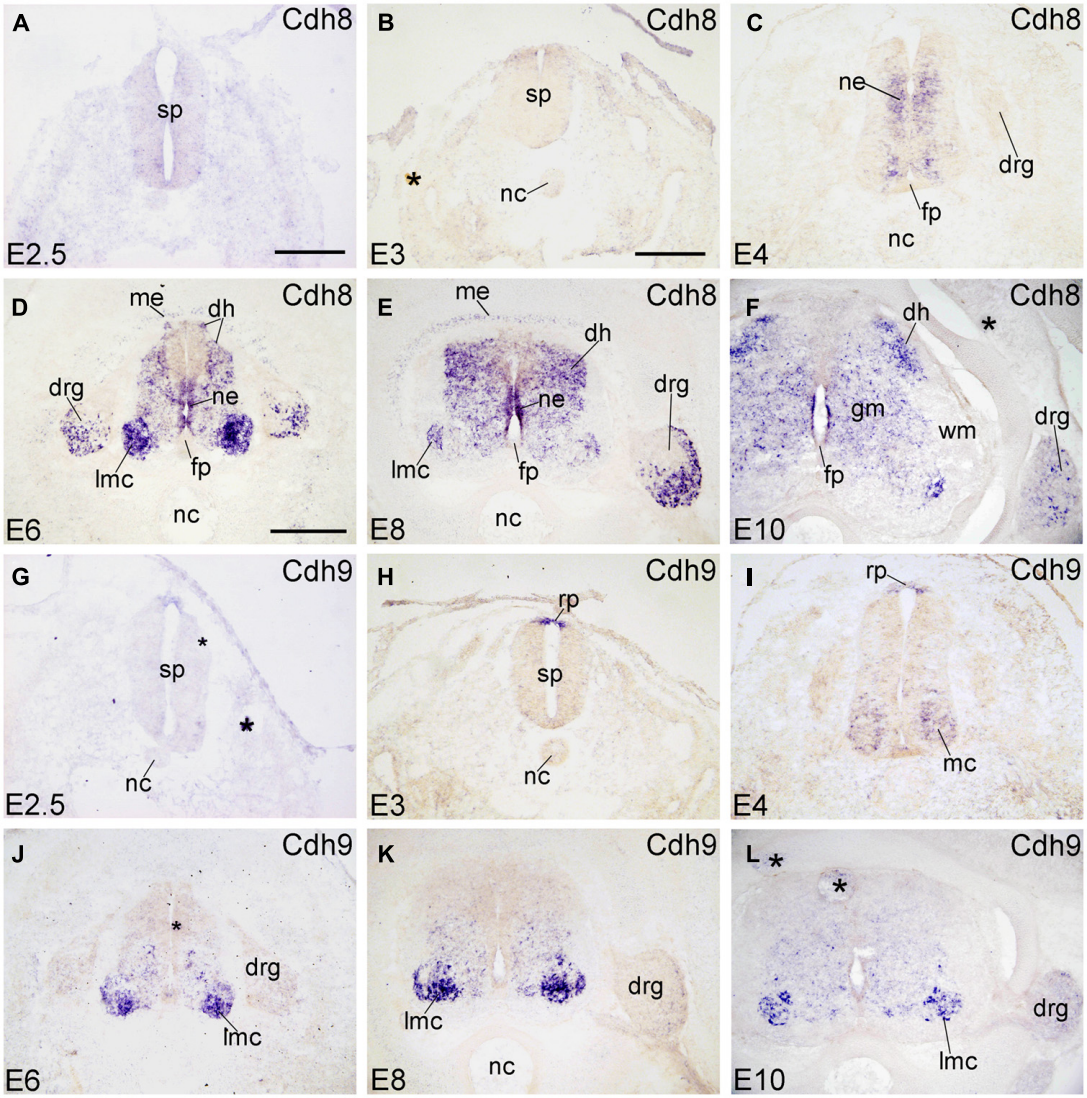
**Expression of Cdh8 and Cdh9 in transverse sections of the developing spinal cord at the lumbar level from 2.5 days’ incubation (E2.5) to E10 (indicated in each panel).** **(A–F)** *In situ* hybridization result for cadherin-8 (Cdh8), and **(G–L)** for cadherin-9 (Cdh9). The asterisks in **B,F,G,J,L** indicate tissue artifacts. Abbreviations: DH, dorsal horn; DRG, dorsal root ganglion; GM, gray matter; LMC, lateral motor column; MC, motor column; ME, meninges; ML, mantle layer; NC, notochord; NE, neuroepithelial layer; RP, roof plate; WM, white matter. Scale bars: 100 μm in **A** (for **A,G**); 200 μm in **B** (for **B,C,H,I**); 500 μm in **D** (for **D–F,J–L**).

**FIGURE 4:**
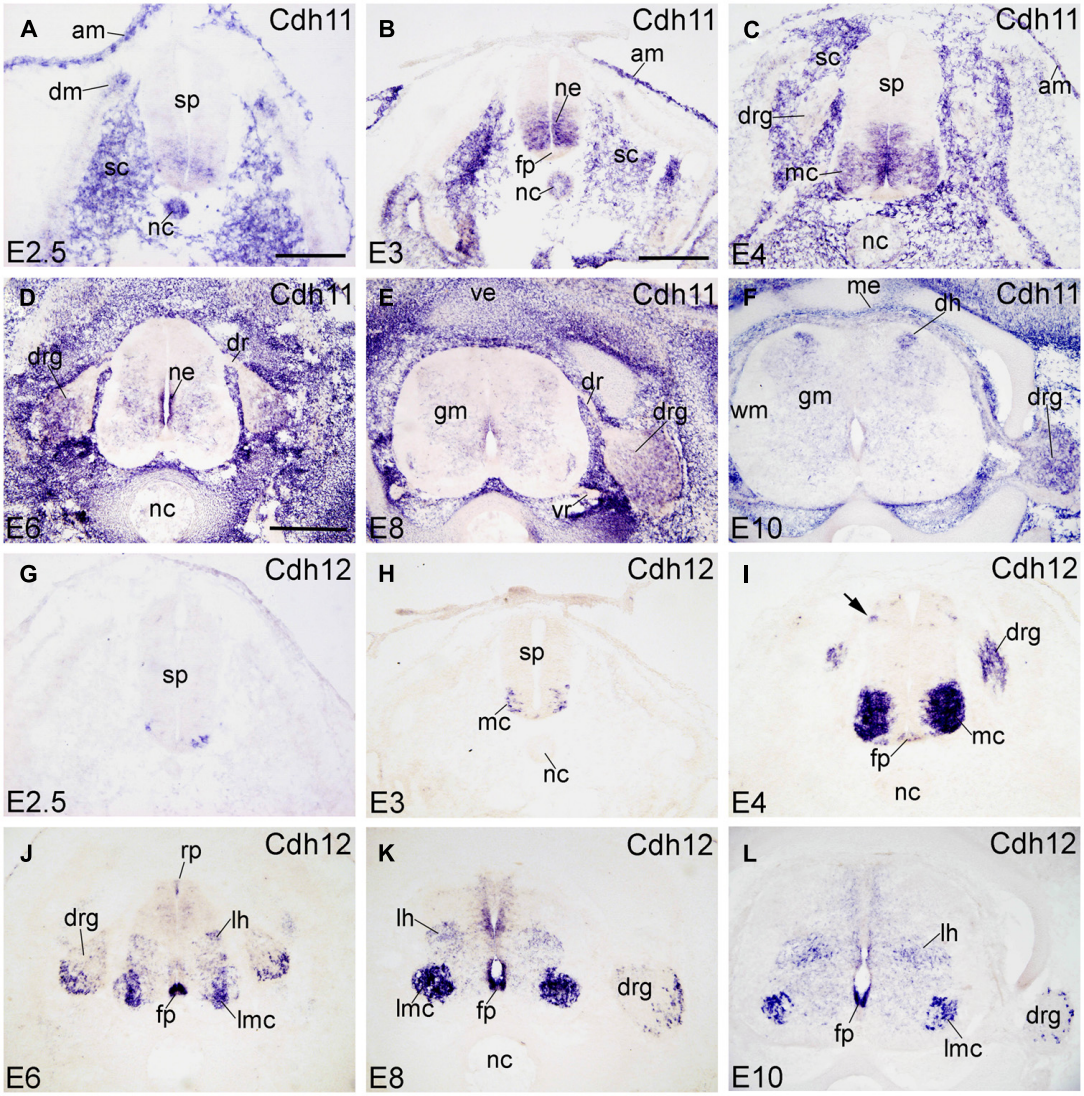
**Expression of Cdh11 and Cdh12 in transverse sections of the developing spinal cord at the lumbar level from 2.5 days’ incubation (E2.5) to E10 (indicated in each panel).** **(A–F)** *In situ* hybridization result for cadherin-11 (Cdh11), and **(G–L) **for cadherin-12 (Cdh12). Abbreviations: AM, amnion; DM, dermomyotome; DR, dorsal root; DRG, dorsal root ganglion; FP, floor plate; GM, gray matter; LH, lateral horn; LMC, lateral motor column; MC, motor column; ME, meninges; ML, mantle layer; NC, notochord; NE, neuroepithelial layer; RP, roof plate; SC, sclerotome; SP, spinal cord; VE, vertebral arch; VR, ventral root; WM, white matter. Scale bars: 100 μm in **A** (for **A,G**); 200 μm in **B** (for **B,C,H,I**); 500 μm in **D** (for **D–F,J–L**).

**FIGURE 5:**
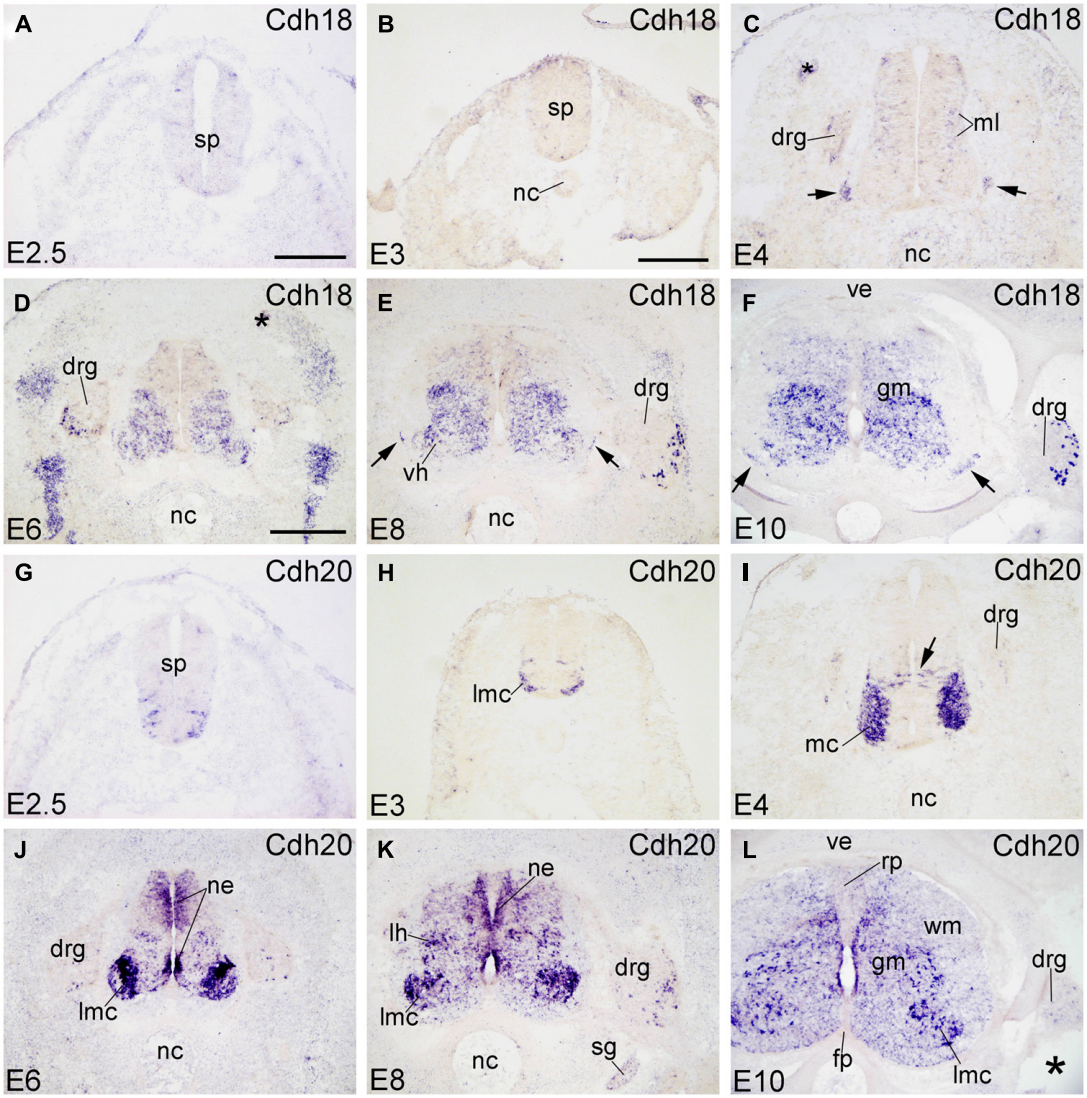
**Expression of Cdh18 and Cdh20 in transverse sections of the developing spinal cord at the lumbar level from 2.5 days’ incubation (E2.5) to E10 (indicated in each panel).** **(A–F)** *In situ* hybridization result for cadherin-18 (Cdh18), and **(G–L) **for cadherin-20 (Cdh20). The arrows in **C,E,F** point to the positive boundary cap cells at the positions of the ventral root. The asterisks in **C,D,L** indicate tissue artifacts. Abbreviations: DM, dermomyotome; DRG, dorsal root ganglion; FP, floor plate; GM, gray matter; LMC, lateral motor column; MC, motor column; ML, mantle layer; NC, notochord; NE, neuroepithelial layer; RP, roof plate; SG, sympathetic ganglion; SP, spinal cord; VE, vertebral arch; VH, ventral horn; WM, white matter. Scale bars: 100 μm in **A** (for **A,G**); 200 μm in **B** (for **B,C,H,I**); 500 μm in **D** (for **D–F,J–L**).

**FIGURE 6:**
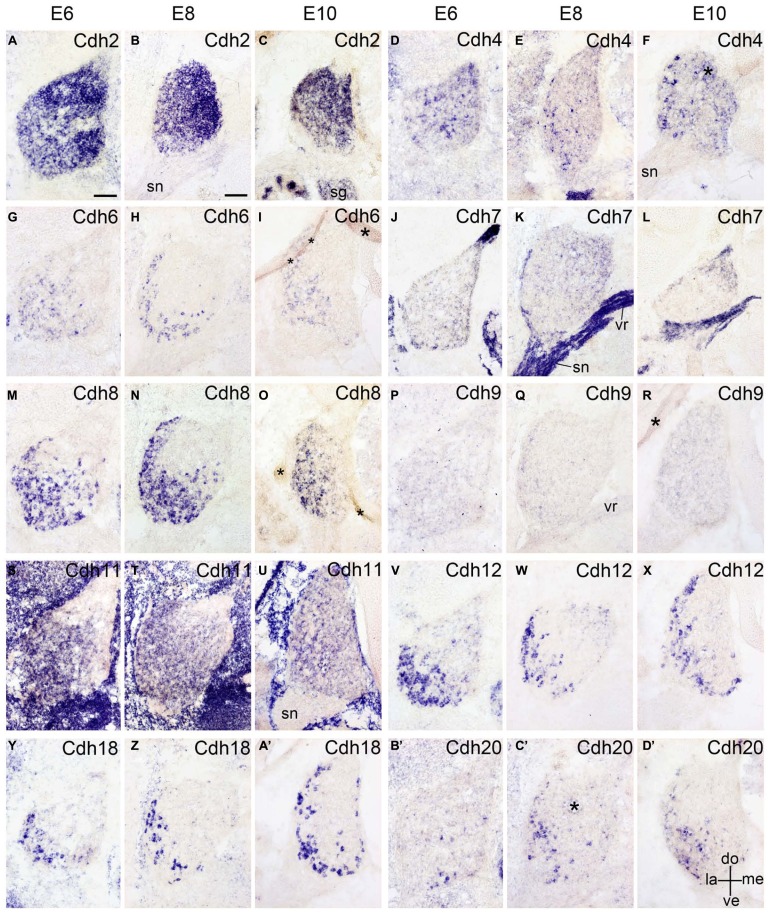
**Expression of Cdh2 (A–C), Cdh4 (D–F), Cdh6 (G–I), Cdh7 (J–L), Cdh8 (M–O), Cdh9 (P–R), Cdh11 (S–U), Cdh12 (V–X), Cdh18 (Y–A’), and Cdh20 (B’–D’) in transverse sections of the developing dorsal root ganglion at the lumbar level from 6 days’ incubation (E6) to E10 (as indicated at the top of the figure for the appropriate vertical rows, respectively).** All panels show *in situ* hybridization results for different classic cadherins (labeled in each panel). The asterisks in **F,I,O,R,C’ **indicate tissue artifacts. Abbreviations: DO, dorsal; LA, lateral; ME, medial; SG, sympathetic ganglion; SN, spinal nerve; ve, ventral; VR, ventral root. Scale bars: 100 μm in **A** (for E6 panels); 200 μm in **B** (for E8–E10 panels).

**FIGURE 7:**
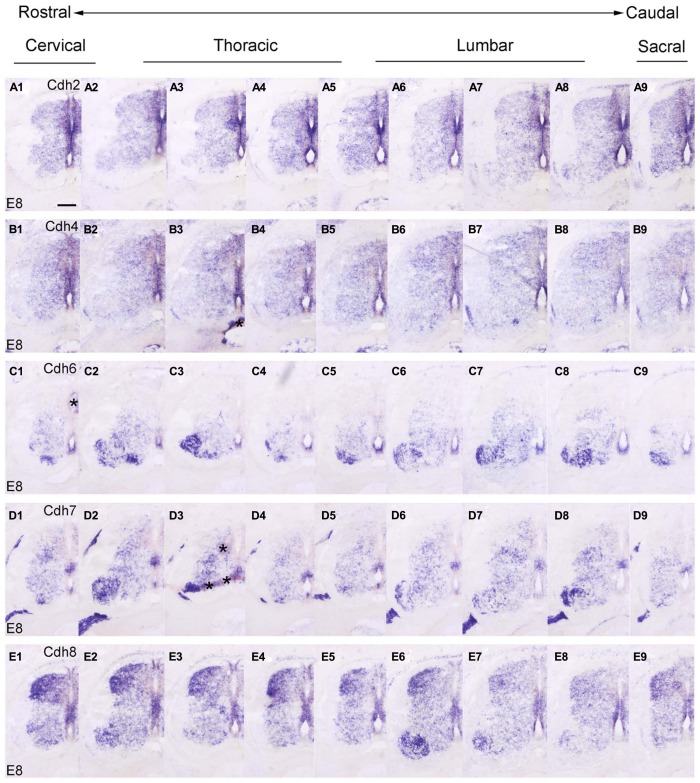
**Rostrocaudal expression profiles of Cdh2 (A1–9), Cdh4 **(B1–9)**, Cdh6 **(C1–9)**, Cdh7 **(D1–9)**, and Cdh8 **(E1–9)** in transverse adjacent sections of the spinal cord at 8 days’ incubation (E8) visualized by *in situ* hybridization.** The asterisks in **B3,C1,D3** indicate artificial folds in the tissue. Scale bar: 200 μm in **A1** (for all panels).

**FIGURE 8:**
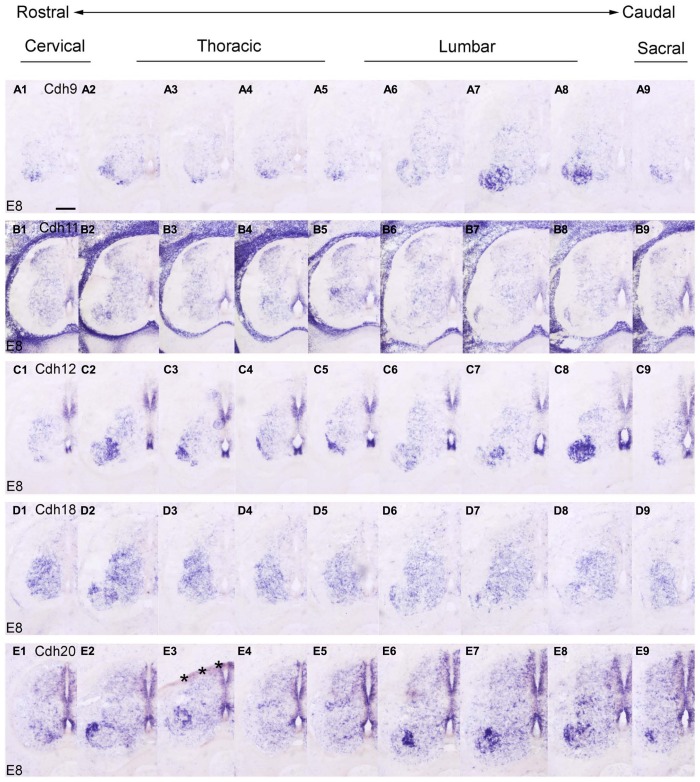
**Rostrocaudal expression profiles of Cdh9 (A1–9), Cdh11 (B1–9), Cdh12 (C1–9), Cdh18 (D1–9), and Cdh20 (E1–9) in transverse adjacent sections of the spinal cord at 8 days’ incubation (E8) visualized by *in situ* hybridization.** The asterisks in **E3 **indicate an artificial fold in the tissue. Scale bar: 200 μm in **A1** (for all panels).

**FIGURE 9:**
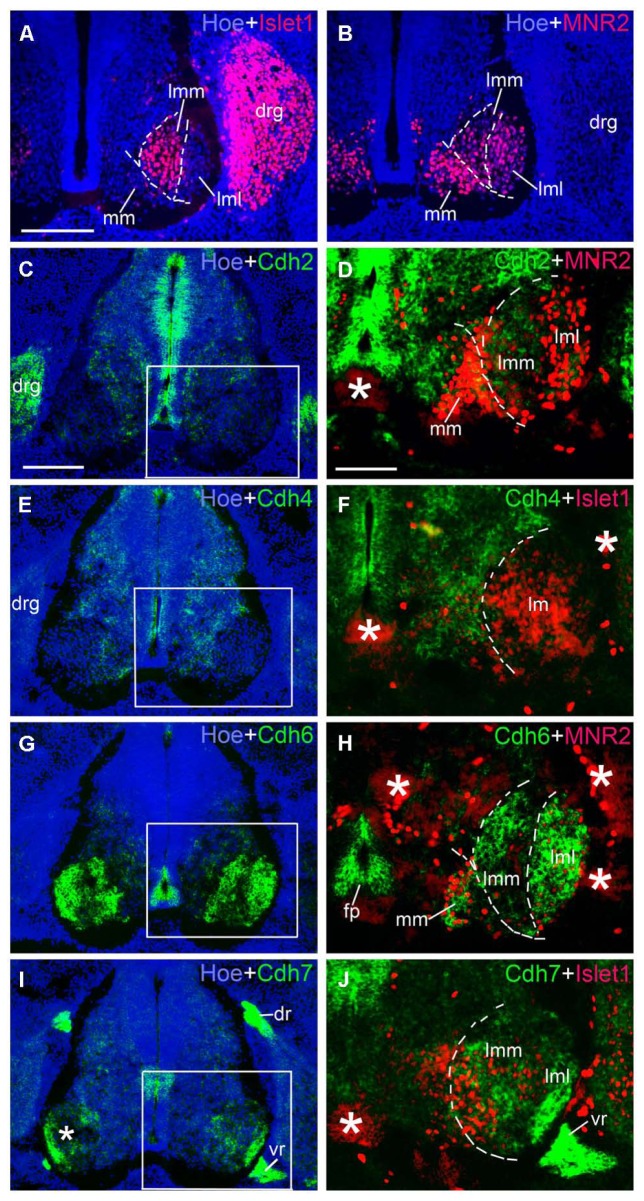
**Expression of Cdh2 (C,D), Cdh4 (E,F), Cdh6 (G,H), Cdh7 (I,J) in motor neuron pools of the spinal cord at the lumbar level at 6 days’ incubation (E6).** Green color represents mRNA signal of individual classic cadherins, as indicated. Islet1 (red color in **A,F,J)** and MNR2 (red color in **B,D,H**) serve as motor neurons markers **(A,B)**. Hoechst 33258 staining visualizes nuclei (Hoe; blue color in **A–C,E,G,I**). The areas boxed in **C,E,G,I** are shown at a higher magnification in **D,F,H,J**, respectively. The dashed lines in **A,B,D,F,H,J **outline the different motor neuron pools. All panels are merged images for different Cdhs, Islet1 or MNR2, and Hoechst staining, respectively (as indicated in each panel). The asterisks in **D,F,H–J** indicate tissue artifacts. Abbreviation: BC, boundary cap cells; DR, dorsal root; DRG, dorsal root ganglion; FP, floor plate; LMC, lateral motor column; lmL, lateral lm; lmM, medial lm; MMC, medial motor column; VR, ventral root. Scale bars: 100 μm in **A** (for **A,B**, in **D** for **F,H,J**); 200 μm in **C** (for **C,E,G,I**).

**FIGURE 10:**
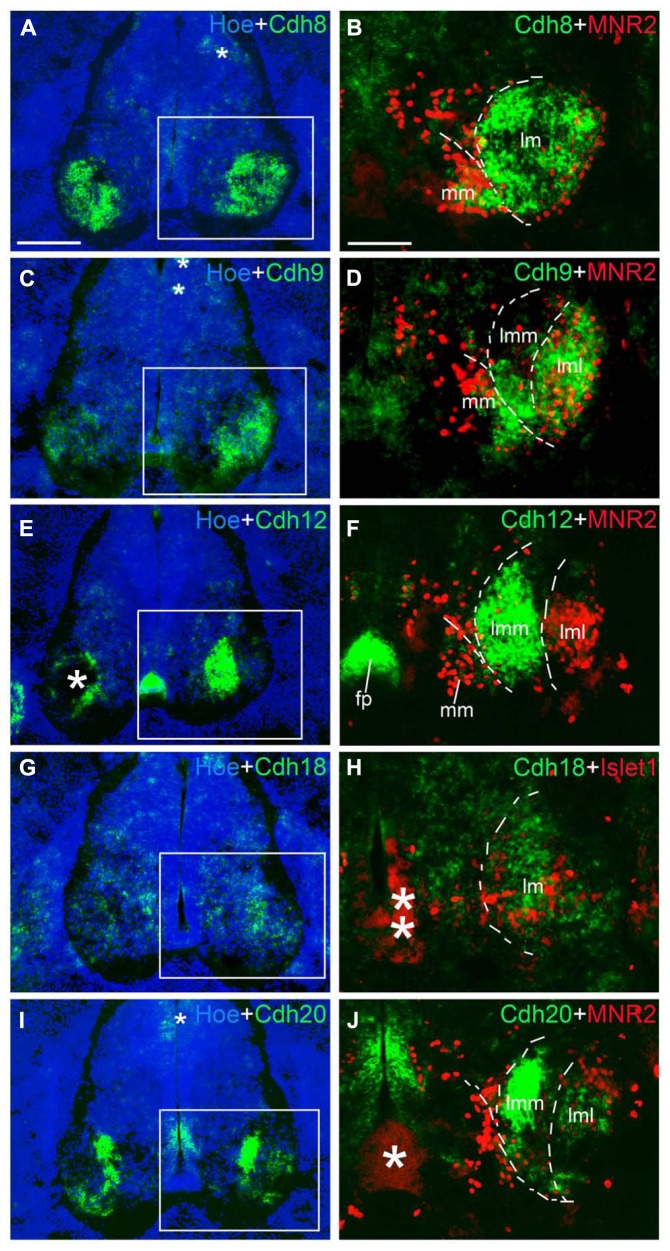
**Expression of Cdh8 (A,B), Cdh9 (C,D), Cdh12 (E,F), Cdh18 (G,H), Cdh20 (I,J) in motor neuron pools of the spinal cord at the lumbar level at 6 days’ incubation (E6).** Green color represents mRNA signal of individual classic cadherins, as indicated. Hoechst 33258 staining visualizes nuclei (Hoe; blue color in **A,C,E,G,I**). The areas boxed in **A,C,E,G,I** are shown at a higher magnification in **B,D,F,H,J**, respectively. The dashed lines in **A,B,D,F,H,J** outline the different motor neuron pools. All panels are merged images for different Cdhs, Islet1 or MNR2, and Hoechst staining, respectively (as indicated in each panel). The asterisks in **A,C,E,H–J** indicate various artifacts in the tissues. Abbreviation: FP, floor plate; LMC, lateral motor column; lmL, lateral lm; lmM, medial lm; MMC, medial motor column. Scale bars: 100 μm in **B** (for **B,D,F,H,J**); 200 μm in **A** (for **A,C,E,G,I**).

Results are shown in the figures in the following sequence. First, the expression patterns of the 10 investigated classic cadherins are sequentially displayed for each cadherin from E2.5 to E10 at the lumbar level of the SP (**Figures 1–5**). **Figure 6** shows the distribution of each cadherin in the DO root ganglion (DRG) at E6, E8, and E10. The panels for each cadherin are arranged according to the developmental stages (early to late). Second, the rostrocaudal expression profiles of the cadherins from cervical to sacral levels of the SP are shown for E8 (**Figures 7** and **8**). Third, **Figures 9** and **10** illustrate cadherin expression by motor neuron pools in sections that were doubly stained for cadherins and a motor neuron marker (Islet1 or MNR2).

### DEVELOPMENTAL EXPRESSION PROFILES AT THE LUMBAR LEVEL (E2.5–E10)

#### Cdh2

Chicken Cdh2 (*N*-cadherin) was identified by Hatta and Takeichi (1986). Cdh2 function is required for retinal axon outgrowth (Matsunaga et al., 1988), the pathfinding of tectofugal axons (Treubert-Zimmermann et al., 2002) and to sort spinal projection axons into spinocerebellar tracts (Sakai et al., 2012). Confirming previous results by Hatta et al. (1987), Cdh2 is strongly expressed in the entire SP neuroepithelium (NE), in the notochord (NC) and in the dermomyotome (DM in **Figures 1D–F**) from E2.5 to E4. It is also detected in the lung bud at E3 (lb in **Figure 1E**). From E4 to E6, Cdh2 mRNA signal is still strong in the NE and moderate in the ML of the SP (ml in **Figure 1G**). As development proceeds, Cdh2 expression becomes weaker in the NC (in **Figures 1F,G**). At E8 and E10 (**Figures 1H,I**), strong signal is still detected in the NE, but signal becomes weaker in the roof plate (RP) and the floor plate (FP); it is no longer observed in the NC (**Figures 1H,I**). Similar to rat Cdh2 (Ncdh; Wanner et al., 2006), chicken Cdh2 is expressed also by the ventral boundary cap (BC) cells (arrows in **Figure 1H**). Interestingly, Cdh2 is expressed in the entire DRG, but more strongly in the dorsomedial than in the ventrolateral part (**Figures 1F,I** and **6A–C**).

#### Cdh4

Cdh4 (*R*-cadherin) was first isolated from chicken retina and called retinal cadherin (Inuzuka et al., 1991a). It shows an expression pattern different from Cdh2 in the neural tube and the peripheral ganglia (Inuzuka et al., 1991b; Redies et al., 1992). At E2.5 and E3, Cdh4 is not expressed in the SP, but in the NC and DM (in **Figures 1J,K**). At E4, Cdh4 is expressed moderately in the ventromedial part of the SP, and strongly in the NC and the DM. It is also detected in the DRG (in **Figure 1L**). From E6–E10, Cdh4 expression becomes moderate throughout the GM of the SP, but signal is absent in the motor neuron pools (**Figures 1M–O**). At E8 and E10, Cdh4 is strongly expressed in the sympathetic ganglion (SG). A few positive cells can be observed in the DRG, (**Figures 6D,E**) as well. Similar to Cdh2, Cdh4 is expressed by ventral BC cells (arrows in **Figures 1N,O**). Unlike Cdh2, Cdh4 remains positive in the NC until E10 (**Figure 1O**); it is the only molecule among the 10 cadherins studied that is still expressed in the NC at late stages. Note that Cdh4 remains strongly positive in the DO region of the DM (in **Figure 1M**), similar to results at the protein level (Inuzuka et al., 1991b).

#### Cdh6

Cdh6 is called cadherin-6B in chicken and has also been named fetal kidney-cadherin in rat (Xiang et al., 1994). At early stages (E2.5–E4), Cdh6 is strongly expressed in the neural crest and the RP (in **Figure 2A**), as demonstrated previously by Nakagawa and Takeichi (1998) and Ju et al. (2004). At E3, the FP also expresses Cdh6 strongly (FP in **Figure 2B**). At E4, Cdh6 is detected also in the MC neuron, with expression being stronger in the medial part than in the lateral part of the MC (**Figure 2C**; Padilla et al., 1998). Cdh6 signal is also detected in the spinal nerve (SN) at this stage (SN in **Figure 2C**). From E6 to E10, Cdh6 is mostly expressed in the basal plate of the SP, with extremely strong signal observed in the LMC neuron (in **Figures 2D–F**). Cdh6 signal is still strong in the FP, but in the RP, Cdh6 expression becomes weaker and is no longer detected at E10 (**Figures 2D–F**). Cdh6 is expressed in the DO ME (ME in **Figures 2D,E**), but no longer at E10 (**Figure 2F**). In the DRG, Cdh6 is detected mainly in scattered cells of its lateroventral portion (**Figures 6G–I**). Unlike Cdh2 and Cdh4, Cdh6 is not expressed in the NC.

#### Cdh7

Cadherin-7 (Cdh7) expression in the chicken SP was described before by Nakagawa and Takeichi (1998) and by our group. For example, it has been reported that Cdh7 is expressed in a radial domain (Nakagawa and Takeichi, 1998) that abuts ventrally the basal/alar plate boundary (Ju et al., 2004). In the SP, sonic hedgehog regulates its expression (Luo et al., 2006). Schwann cells of the SN also express Cdh7 (Nakagawa and Takeichi, 1998; Lin et al., 2010). At E2.5 and E3, Cdh7 is expressed by neural crest cells (NCCs) and by a sharply demarcated dorsoventral neuroepithelial domain of the basal plate (**Figures 2G,H**). At E4, Cdh7 signal is also found in the DRG and the SN (in **Figure 2I**), which derive from the neural crest. From E6 to E10, Cdh7 is moderately expressed in the GM of the SP, but strongly in the LMC neuron (in **Figures 2J–L**). Cdh7 is weakly expressed in the DRG (**Figures 6J–L**). Interestingly, it is very strongly expressed along peripheral nerve fibers, such as the dorsal root (DR), the ventral root (VR) and the SN (in **Figures 2I,K** and **6K**). It is also strongly expressed by the ventral BC cells of the SP (arrows in **Figures 2K,L**), as reported before (Barnes et al., 2010). The dorsal BC cells also express Cdh7 at the position where the DR enters the SP (arrowhead in **Figure 2L**).

#### Cdh8

Cadherin-8 (Cdh8) is expressed in restricted regions of the developing chicken brain (Lin et al., 2008). At early stages of development, Cdh8 is not detected in the SP and its surrounding tissues (E2.5 and E3; **Figures 3A,B**). At E4, Cdh8 signal appears in the NE of the SP at intermediate dorsoventral positions, but not in the innermost ependymal layer (**Figure 3C**). From E6 to E10, Cdh8 expression is detected also in the developing ML, most prominently in the LMC neuron of the VH, but also in the dorsal horn (DH in **Figures 3D–F**). It is detected in the NE of the basal plate, but not of the FP (**Figures 3D–F**). Cdh8 is also expressed in the ME surrounding the dorsal SP (ME in **Figures 3D,E**). In the DRG, Cdh8 signal is restricted to the lateroventral region (**Figures 3D–F** and **6M–O**). Strong expression of Cdh8 in the DH and scattered cells in the GM (**Figures 3E,F**) has also been observed for mouse Cdh8 (Korematsu and Redies, 1997; Suzuki et al., 2007).

#### Cdh9

Cdh9 is also named T1-cadherin in humans. In the chicken SP, Cdh9 expression is detected in the RP at E3 (**Figure 3H**), but not yet at E2.5 (**Figure 3G**). At E4, Cdh9 signal has decreased in the RP but can be detected in the motor neuron column (MC in **Figure 3I**). From E6 to E10, it is prominent in the basal plate of the SP, most strongly in the LMC neuron (in **Figures 3J–L**). RP expression is no longer observed. No signal is detected in the DRG (**Figures 3J–L** and **6P–R**).

#### Cdh11

Cdh11 (also named osteoblast cadherin [OB-cdh] in humans) is expressed in the basal plate of the SP at early developmental stages (E2.5–E4, **Figures 4A–C**). Cdh11 expression in the NC decreases as development proceeds (**Figures 4A–C**) and is not detected after E4 (**Figures 4D–F**). In the surroundings of the SP, Cdh11 is strongly expressed in the sclerotome (SC). It is the only cadherin of the present study that is expressed by the amnion (AM in **Figures 4A–C**). From E6 to E10, Cdh11 expression is prominent in the mesenchymal cells surrounding the SP (**Figures 4D–F**), which confirms Cdh11 as a mesenchymal cell marker (Simonneau et al., 1995). In the SP, Cdh11 is moderately expressed in the GM. In the ventral NE, it is strongly expressed from E3 to E6, then decreases at E8, and is no longer detected at E10; this developmental down-regulation of expression is observed also in the mouse (Padilla et al., 1998). In the DH at E10 (DH in **Figure 4F**), Cdh11 is expressed in a distinct medial region, in a pattern roughly complementary to that of Cdh8 (**Figure 3F**). Cdh11 signal is found also in ventrolateral regions of the DRG and in the mesenchymal cells surrounding the DRG (**Figures 4D–F** and **6S–U**), but not along the SN (in **Figure 6U**).

#### Cdh12

Cdh12 (also named *N*-cadherin-2 or Br-cadherin) is detected only in a subset of cells in the ventrolateral region of the ML at E2.5 and E3 (**Figures 4G,H**). At E4, Cdh12 is strongly expressed in the motor column (MC) neuron, and weakly in the FP and in a DO region of the SP (arrow in **Figure 4I**). Cdh12 is also detected in the DRG at this stage (**Figure 4I**). From E6 to E10, expression becomes strong in the FP (in **Figures 4J–L**); in the RP, it is detected in a few cells at E6 only (RP in **Figure 4J**). Signal is strong also in the LMC neuron , but moderate in an intermediate dorsoventral region (LH, lateral horn; **Figures 4J–L**), and in restricted domains of the NE (**Figures 4J–L**). In the DRG (in **Figures 4J–L**), Cdh12 is expressed in a subset of cells in its lateral region (**Figures 4J–L** and **6V–X**), in a pattern similar to that of Cdh6 (**Figures 2D–F**).

#### Cdh18

Signal of Cdh18 (also called cadherin-14) is not detected at early embryonic stages (E2.5 and E3; **Figures 5A,B**). At E4, Cdh18 expression is scattered in the ML of the SP (ventral region, complementary to the Cdh8 expression pattern) and in the DRG (in **Figure 5C**). From E6 to E10, Cdh18 is moderately expressed in the ventral GM of the SP (**Figure 5E**). In the DRG, it is expressed in scattered large-sized cells in the ventrolateral region (DRG, **Figures 5D–F** and **6Y–A’**). Cdh18 signal is also found in ventral BC cells (arrows in **Figures 5C,E,F**), similar to Cdh2 and Cdh4 (arrows in **Figures 1H,M–O**).

#### Cdh20

Cdh20 (also called MN-cadherin) shares high sequence similarity with Cdh7 and Cdh19 (Kools et al., 2000; Lin et al., 2010). It is expressed in the motor neuron column of the developing SP (Price et al., 2002; Shirabe et al., 2005), where sonic hedgehog regulates its expression (Luo et al., 2009). The present data extend the previous results and demonstrate that, in addition to the motor column (MC) neuron, Cdh20 is expressed in other areas of the developing SP (**Figures 5G–L**). At early stages (E2.5 and E3), Cdh20 signal is detected in the ventrolateral part of the basal plate (**Figures 5G,H**), similar to Cdh12 (**Figures 4G,H**). At E4, Cdh20 is strongly expressed in the MC neuron and in a few cells in the NE (arrow in **Figure 5I**) at a position ventral to the Cdh7-positive radial glia (Luo et al., 2009); these cells may represent radial glial cells or post-mitotic neuroblasts during their migration to the ML. From E6 to E10, Cdh20 is strongly expressed in the LMC neuron, by scattered cells in intermediate dorsoventral domains, and in DO and ventral regions of the NE. No signal can be detected in the RP or the FP. Cdh20 signal is also seen in a few cells of the WM (in **Figure 5L**). In the DRG, a few Cdh20-positive cells are observed in the ventrolateral region (**Figures 5I–L** and **6B’–D’**).

### THE ROSTROCAUDAL EXPRESSION PROFILE OF EACH CLASSIC CADHERIN

In the preceding sections, we described the developmental expression data for the 10 investigated classic cadherins at lumbar levels. Because we observed differences in the rostrocaudal expression profiles of the cadherins, we compared the expression of each cadherin at different rostrocaudal levels (cervical, thoracic, lumbar, and sacral) of the SP. For this purpose, we chose to study an intermediate stage of embryonic development (E8), when different cell groups in the DO and VHs can be discriminated (**Figures 7** and **8**).

At E8, similar to the delta-Pcdhs (Lin et al., 2012), the 10 investigated classic cadherins show distinct expression patterns in the NE, in the GM and in the DR ganglia at all rostrocaudal levels studied. The expression profile for Cdh8 and Cdh11 varies at different rostrocaudal levels of the DH (**Figures 7E1–9** and **8B1–9**). Specifically, the expression of both cadherins is weaker at lumbar and sacral levels (**Figures 7E7,E8** and **8B7,B8**). Interestingly, in the basal plate, striking rostrocaudal differences are observed, especially in the motor pools (**Figures 7** and **8**). For example, Cdh6, Cdh7, Cdh8, Cdh9, Cdh12, and Cdh20 are expressed strongly in specific motor neuron columns, especially at the levels that innervate the limbs (**Figures 7C2,C3,C6–8,D2,D3,D8,E3,E6,E7** and **8A2,A7,A8,C2,C3,C7,C8,E2,E6–8**). Only for Cdh2 (**Figures 7A1-9**) and Cdh4 (**Figures 7B1-9**), expression is weak or absent in the motor neuron pools (**Figures 7A3,A7,A8,B3,B7,B8**). The differential expression of classic cadherins in the motor neuron pools suggests that they are involved in the maturation of the motor pools and in their innervation of specific groups of muscles, in particular in the limbsk (Price, 2012).

### DIFFERENTIAL EXPRESSION PATTERNS OF CLASSIC CADHERINS IN THE MOTOR NEURON POOLS

We next studied the differential expression of classic cadherins in the motor neuron pools in greater detail. Each group of spinal motor neurons projects axons to distinct muscular targets. Along the rostrocaudal dimension, motor neuron that innervate the limb musculature are located in the lower cervical and the lumbar regions of the SP. In the lumbar region of the chicken, motor neurons form two main columns, the MMC neuron and the LMC neuron (Dasen and Jessell, 2009; Lin et al., 2012; Price, 2012). Within the lm, two sub-columns can be discerned: medial lm (lmM) neurons, which innervate ventral limb muscles, and lateral lm (lmL) neurons, which innervate DO limb muscles (William et al., 2003). We identified these different motor columns (MCs) with the motor neuron markers Islet1, a Lim homeodomain protein, and MNR2, a member of an evolutionarily conserved subgroup of Mnx class proteins. Double staining with these markers was carried out in the lumbar SP at E6 (**Figures 9** and **10**) and E9 (data not shown). Islet1 is strongly expressed in the lmM but weakly in the MM (Pfaff et al., 1996; **Figure 9A**). MNR2 signal is strong in the MM but more weak in the lm (Ferrier et al., 2001; **Figure 9B**).

Note that, in the double-labeled sections, strong *in situ* hybridization signal for cadherins (NBT/BCIP precipitates) can obscure weak (fluorescent) immunostaining signal for Islet1 and MNR2. This quenching may result in the false impression of an absence of Islet1 and MNR2 signal in some areas of co-expression with cadherins in **Figures 9** and **10**. Nevertheless, by comparison with the single-label results for Islet1 and MNR2 (**Figures 9A,B**), the different motor neuron pools can be identified clearly.

**Table 2** summarizes the results for the motor neuron pools. A comparison with the motor neurons markers demonstrates the paucity of Cdh2 and Cdh4 expression in the motor neuron pools (**Figures 9C–F**). Cdh6 is detectable both in the MM and the lm, and there is a clear boundary between the lmM and the (lmL; **Figures 9G,H**). Cdh7 is expressed in some cell clusters in the lm, but not in the MM (**Figures 9I,J**); expression is also strong in the DR and the (VR; **Figure 9J**). Similar to Cdh6, Cdh8 is strongly expressed in the MM and the lm (**Figures 10A,B**). Interestingly, Cdh9 signal is found in the mm, but regionally expressed in the lm, and it is much weaker in the lmM than in the lmL (**Figures 10C,D**). In contrast, Cdh12 expression is prominent only in the lmM, but not in the MM and the lmL (**Figures 10E,F**); it is also strongly expressed in the FP (**Figure 10F**). Compared to other cadherins, Cdh18 expression is weak and regionally detected in the scattered cells in the MM and the lm (**Figures 10G,H**). As motor neuron cadherin (MN-cadherin), Cdh20 is expressed in the lm, where it is stronger in the lmM than in the lmL; Cdh20 signal is largely absent in the MM (**Figures 10I,J**).

**Table 2:** Expression of classic cadherins in the different motor neuron pools.

	MMC	lmM	lmL
Cdh2	-	-	-
Cdh4	-	-	-
Cdh6	+	++*	+++
Cdh7	-	+*	+++*
Cdh8	+	++	++
Cdh9	++	+*	++
Cdh11	-	-	-
Cdh12	-	+++	-
Cdh18	-	+	+
Cdh20	-	++*	+*

-, negative; +, weak expression; ++, moderate expression; +++, strong expression; * indicates that the cadherin is partially expressed in the motor neuron pool (for example, Cdh6 is moderately and partially expressed in the medial lm). Abbreviations: Cdh, cadherin; LM, lateral motor neuron pool; lmL, lateral lm; lmM, medial lm; MM, medial motor neuron pool.

## DISCUSSION

Cadherins play an important role in the morphogenesis and functional differentiation of the nervous system (Redies et al., 1993, 2011; Redies, 2000; Takeichi, 2007; Barnes et al., 2010; Price, 2012) and in nervous system diseases (Agiostratidou et al., 2007; Price, 2012; Redies et al., 2012). The detailed analysis of cadherin expression patterns allows deriving well-founded hypotheses about the role of individual cadherins in nervous system development and mature CNS function. In the present study, we mapped the expression of 10 members of the classic cadherins (Cdh2, Cdh4, Cdh6, Cdh7, Cdh8, Cdh9, Cdh11, Cdh12, Cdh18, and Cdh20) in the developing chicken SP and its surrounding tissues. Extending previous studies on individual classic cadherins, we provide more detailed expression data and compare multiple classic cadherins in the developing SP. Similar to our previous results on delta-Pcdhs (Lin et al., 2012), the present results indicate that each cadherin has unique (but partially overlapping) expression pattern that is under tight spatiotemporal control during SP development. In the following sections, the spatiotemporal expression profiles and the potential roles of classic cadherins during SP development will be discussed in detail.

### DORSOVENTRAL PATTERNING OF CADHERIN EXPRESSION IN THE NEUROEPITHELIAL LAYER

The signaling molecules that are secreted from the FP and the RP, such as Shh, Wnts and bone morphogenetic proteins, provide dorsoventral positional information to neural progenitor cells in the NE of the early embryonic SP (Wilson and Maden, 2005; Ulloa and Martí, 2010; Le Dréau and Martí, 2012). Our results show that most of the classic cadherins have distinct expression domains along the dorsoventral dimension in the NE during SP morphogenesis (**Figures 1–5**). An exception is Cdh2, which is strongly expressed throughout the entire NE from early stages of its formation, similar to its expression in the brain (Redies et al., 1993). In parallel to the relative thinning of the NE during development, the neuroepithelial expression domains of the classic cadherins become narrower. The domains keep their dorsoventral position (**Figures 1D–I**), similar to delta-Pcdhs (Lin et al., 2012). The onset of expression in the NE varies for different cadherins. For example, Cdh7 and Cdh11 are already expressed in the NE at very early stages. As development proceeds, their expression becomes weaker and finally disappears from the NE. In the ventricular layer of the basal plate, Cdh11 signal is located more ventrally than the Cdh7 signal (**Figures 2G–L** and **4A–F**), which coincides with the dorsalmost progenitor domain of the basal plate in the chicken SP (**Figures 2G,H**; Ju et al., 2004; Lin et al., 2012). Cdh4, Cdh6, Cdh8, Cdh12, and Cdh20 are expressed in different dorsoventral domains of the NE, but their expression starts at later stages (detectable at or after E4). For example, Cdh6 is positive only in the FP and a small adjacent area (**Figures 2C–F**). Strikingly, similar to delta-Pcdh18 (Lin et al., 2012), Cdh12 (**Figures 4K,L**), and Cdh20 (**Figures 5J–L**) have multiple dorsoventral domains of expression, suggesting that their expression does not depend on a singular combination of dorsoventral positional signals. Only two of the investigated classic cadherins, Cdh9 (**Figures 3A–F**) and Cdh18 (**Figures 5A–F**), are totally absent from the NE.

Experimental evidence suggests that a change of cadherin expression at a border between progenitor domains serves to stabilize the border by localizing cells to a specific progenitor domain (Espeseth et al., 1998; Inoue et al., 2001). Whether the classic cadherins have similar functions in the chicken SP remains to be established.

### REGIONAL EXPRESSION OF CADHERINS IN THE SPINAL CORD GRAY MATTER

Spatiotemporally restricted expression patterns are observed for classic cadherins in the GM of the embryonic SP along all three dimensions (dorsoventral, mediolateral, and rostrocaudal) from the beginning of ML formation, when the first post-mitotic neuroblasts arrive in the incipient ML. Similar findings were obtained previously for delta-Pcdhs in the SP (Lin et al., 2012) and for many cadherins in the developing brain (Redies et al., 1993, 2012; Redies, 2000; Takeichi, 2007; Lin et al., 2008; Krishna-K et al., 2009; Hertel and Redies, 2011; Hertel et al., 2012; Matsunaga et al., 2013).

For example, Cdh7 signal assumes a heterogeneous expression in mediolateral and dorsoventral directions in the GM (**Figures 2J,K**). Along the rostrocaudal axis, Cdh7 expression is observed in motor neurons that are linked to limb muscles (Price et al., 2002). Cdh9 signal is almost exclusively restricted to the LMC. Cdh8 and Cdh18 show complementary expression patterns in the ML at E4. At the lumbar level, all investigated classic cadherins, except for Cdh9 and Cdh11, are expressed in the ML; their expression patterns differ from E6 onward. In the DH, Cdh6, Cdh7, Cdh12, and Cdh20 are strongly expressed and Cdh8 and Cdh11 show opposing mediolateral gradients of expression.

A focus of the present investigation was placed on the MC where striking differences between the expression profiles of individual classic cadherins can be observed. Our results show that, except for Cdh2, Cdh4 and Cdh11, the classic cadherins are prominently expressed only at the cervical and lumbar levels (**Figures 7** and **8**), from where the limbs are innervated. Differential expression is prominent along the mediolateral dimension in the MC from E6 to E10 (**Figures 1–5, 9**, and **10**). The expression of several cadherins is heterogeneous also in the different pools of motor neurons. For example, Cdh9 is expressed weakly in the medial part of the LMC, but strongly in the lateral part of it (lmL, **Figures 10C,D**), while the inverse pattern is observed for Cdh12 (**Figures 10E,F**). Some cadherins are also subject to temporal regulation of expression in the motor neuron pools. For example, Cdh8 is strongly expressed in the LMC at E6, but becomes weaker at E8 and E10 (**Figures 3D–F**). In the mouse, Cdh11 is expressed in the VH where its expression is spatiotemporally regulated in a way complementary to Cdh6, suggesting different functions in motor neuron differentiation (Marthiens et al., 2002). Our results in chicken corroborate findings in mice that Cdh11 expression sets in at an early stage (before E4, **Figures 4A–C**) and decreases to low levels from E6 onward (**Figures 4D–F**) when Cdh6 signal is still prominent (**Figures 2D–F**; Marthiens et al., 2002).

The present expression patterns in the VH are compatible with the notion that classic cadherins play a role in functional neuronal organization, as demonstrated previously at the experimental level. For example, it has been shown that some classic cadherins regulate the sorting of motor neuron pools in the chicken SP (Price et al., 2002). The finding that spinal motor neuron migration and pattern formation requires γ-catenin-dependent cadherin function suggests a prolonged role for cadherin expression in all phases of motor neuron organization (Bello et al., 2012).

More generally, classic cadherins play various other roles during functional CNS differentiation, for example in axon guidance and targeting. Cdh2, Cdh4, Cdh6, and Cdh7 regulate the sorting of axons to different tectofugal pathways (Treubert-Zimmermann et al., 2002). Cdh2 inhibition mediated by Robo controls the spinal projection and axon sorting into the spinocerebellar tract (Sakai et al., 2012). Slit-Robo signaling downregulates Cdh2 activity to allow apical retraction in newly generated retinal ganglion cells (Wong et al., 2012). Cdh6 and Cdh7 not only differ in their expression profiles (**Figure 2**; Ju et al., 2004), but also in how they regulate the growth, branching and guidance of cranial motor axons (Barnes et al., 2010). Mouse Cdh2 and Cdh8 both are critical to generate the hippocampal mossy fiber pathway; the two molecules complement each other in the assembly of the synaptic circuit and differentially contribute to afferent and target differentiation (Bekirov et al., 2008). In the developing hippocampus, mouse Cdh9 can regulate synapse-specific differentiation (Williams et al., 2011). These examples illustrate some of the roles, which the spatiotemporal cadherin expression patterns play in CNS development (for a comprehensive review, see Hirano and Takeichi, 2012).

### EXPRESSION OF CADHERINS IN PERIPHERAL GANGLIA

The peripheral ganglia, such as the DRG and the SG, are derived from the neural crest (Kasemeier-Kulesa et al., 2005). The DRG contains different populations of long-distance projecting neurons that transmit signals from various sensory organs toward the appropriate integration center (Rozanski et al., 2013). In this study, except for Cdh9, all investigated classic cadherins are expressed in the DRG of the chicken embryo, but in different spatiotemporal distributions (**Figure 6**) similar to those of delta-Pcdhs (Lin et al., 2012). For example, Cdh2 is expressed throughout the DRG in a dorsomedial gradient from E4 onward (**Figures 1F,I** and **6A–C**). Neuronal firing in the mouse regulates Cdh2 in cultured DRG neurons (Itoh et al., 1997). Cdh4 is only expressed in a few scattered cells in the DRG (**Figures 6D–F**). Interestingly, Cdh6, Cdh8, Cdh11, Ch12, Cdh18, and Cdh20 are all expressed in the lateroventral region of the developing DRG (**Figure 6**), but the cell size and the exact distribution of the different cadherin-expressing neuron populations are not identical. These results suggest that classic cadherins reflect the intrinsic heterogeneity of DRG cell types (Arber et al., 2000; Kramer et al., 2006; Montelius et al., 2007). It remains to be studied whether the cadherin-expressing populations overlap with known subpopulations or with the subpopulations that express delta-Pcdhs (Lin et al., 2012). The sympathetic ganglia are positive for Cdh2 and Cdh4, but not for the other classic cadherins (**Figures 1H,N,O**). The roles of classic cadherins in the developing peripheral ganglia remain to be studied.

### EXPRESSION OF CADHERINS BY THE BOUNDARY CAP CELLS AND MENINGES

The BC cells are located at the exit points of the motor and sensory roots from the SP. They are neural crest-derived stem cells, which participate in the formation of the boundary of the central/peripheral nervous system and can be redirected into CNS lineages (Zujovic et al., 2011). The ventral BC cells express Cdh2, Cdh4, Cdh7, and Cdh18 (**Figures 1H,M–O** and **5F**). Cdh7 signal is also found in the dorsal BC cells (**Figures 2K,L**). In the rat embryo, Cdh2 expression in the BC cells is restricted to the period of axon outgrowth (Wanner et al., 2006). Ablation of BC cells results in the ectopic location of motor neurons in the peripheral nervous system, which suggest that BC cells regulate motor neuron location and axon outgrowth at the motor exit point (Bron et al., 2007). The co-expression of cadherins by motor neurons and BC cells suggest that the BC cells allow motor nerve outgrowth by providing a homophilic adhesive substrate, as proposed previously for Cdh2 (Wanner et al., 2006).

Cdh6, Cdh8, and Cdh11 are three members of the 10 classic cadherins expressed in the ME around the SP. Whereas signal for Cdh11 is ubiquitous (**Figures 4D–F**), Cdh6 (**Figures 2D,E**) and Cdh8 (**Figure 3E**) are expressed in the dorsal ME only, similar to Pcdh8 (Lin et al., 2012).

### EXPRESSION IN THE DERMOMYOTOME, SCLEROTOME, AND NOTOCHORD

At E2.5–E6, Cdh2 and Cdh4 are expressed in the DM, which gives rise to the trunk and limb muscles during embryonic development. At E2.5 only, Cdh6 and Cdh11 are detected in the DO part of the DM. In the SC, which contributes to the bones, including the vertebrae, Cdh7 is strongly expressed at E2.5 and E3. Cdh11 is widely expressed in the mesenchymal cells of the SC at E2.5–E6, suggesting its association with mesenchymal morphogenesis of the trunk (Kimura et al., 1995).

The NC is a transient mesodermal structure with patterning functions in the early vertebrate embryo. Cdh4 expression is prominent in the NC until at least at E10 (Inuzuka et al., 1991b). At earlier stages, the NC is also strongly positive for Cdh2 and Cdh11. The NC secretes the key morphogen sonic hedgehog homolog (SHH), which plays a role in patterning the ventral SP (Echelard et al., 1993) and regulates the expression of Cdh7 and Cdh20 (Luo et al., 2006, 2009). It is at present unclear whether SHH signaling regulates also the patterned expression of the other classic cadherins.

## AUTHOR CONTRIBUTIONS

Juntang Lin planned and carried out the experiments, analyzed the data and wrote the manuscript, Congrui Wang attended part of the experiments, and Christoph Redies supervised the project and contributed to writing the manuscript.

## Conflict of Interest Statement

The authors declare that the research was conducted in the absence of any commercial or financial relationships that could be construed as a potential conflict of interest.
